# The mathematics of tanning

**DOI:** 10.1186/1752-0509-3-60

**Published:** 2009-06-09

**Authors:** Josef Thingnes, Leiv Øyehaug, Eivind Hovig, Stig W Omholt

**Affiliations:** 1 Centre for Integrative Genetics (CIGENE), Norwegian University of Life Sciences (UMB), PO Box 5003, 1432 Ås, Norway; 2 Department of Mathematical Sciences and Technology, Norwegian University of Life Sciences (UMB), PO Box 5003, 1432 Ås, Norway; 3 Department of Animal and Aquacultural Sciences, Norwegian University of Life Sciences, PO Box 5003, 1432 Ås, Norway; 4 Department of Tumor Biology, Institute for Cancer Research, Norwegian Radium Hospital, Montebello, 0310 Oslo, Norway; 5 Department of Medical Informatics, Norwegian Radium Hospital, Montebello, 0310 Oslo, Norway; 6 Department of Informatics, University of Oslo, PO Box 1080 Blindern, 0316 Oslo, Norway

## Abstract

**Background:**

The pigment melanin is produced by specialized cells, called melanocytes. In healthy skin, melanocytes are sparsely spread among the other cell types in the basal layer of the epidermis. Sun tanning results from an UV-induced increase in the release of melanin to neighbouring keratinocytes, the major cell type component of the epidermis as well as redistribution of melanin among these cells. Here we provide a mathematical conceptualization of our current knowledge of the tanning response, in terms of a dynamic model. The resolution level of the model is tuned to available data, and its primary focus is to describe the tanning response following UV exposure.

**Results:**

The model appears capable of accounting for available experimental data on the tanning response in different skin and photo types. It predicts that the thickness of the epidermal layer and how far the melanocyte dendrites grow out in the epidermal layers after UV exposure influence the tanning response substantially.

**Conclusion:**

Despite the paucity of experimental validation data the model is constrained enough to serve as a foundation for the establishment of a theoretical-experimental research programme aimed at elucidating the more fine-grained regulatory anatomy underlying the tanning response.

## Background

Around 1 million years ago, a tanning response evolved in our hominid ancestors in which the accumulation of melanin granules in skin cells provided physical protection against the DNA-damaging effects of sunlight [1]. Today the tanning response is exploited by millions of people each year for cosmetic reasons. Because of the increased risks for melanoma and squamous cell carcinoma following overexposure to sunlight [2], the molecular biology of the tanning response has been given substantial biomedical attention over the last decades from dermatologists and oncologists (reviewed by [3-6]), as well as from those seeking ways to achieve tanning independent of sunlight [7].

The biomedical importance of the tanning response, and the potential benefits associated with being able to induce the response in safe ways, call for the establishment of deep knowledge of the underlying regulatory anatomy. However, despite some promising progress in recent years our understanding of the tanning response as a complex process in a system dynamics context is still rather moderate.

It is a common experience that the regulatory anatomy of complex biological systems involving several actors and intricate feedback relationships can be very hard to understand in qualitative as well as quantitative terms without guidance from a mathematical conceptualization of the dynamics. Many conceive that mathematical models are of no use until enough data are available so that they can be made very detailed. However, the heuristic importance of simple models should not be underestimated, as they serve as very efficient interfaces between various disciplines and help us to assess whether our current conceptions of mechanisms, processes and interactions do really lead to the dynamic behaviours we observe.

**Figure 1:**
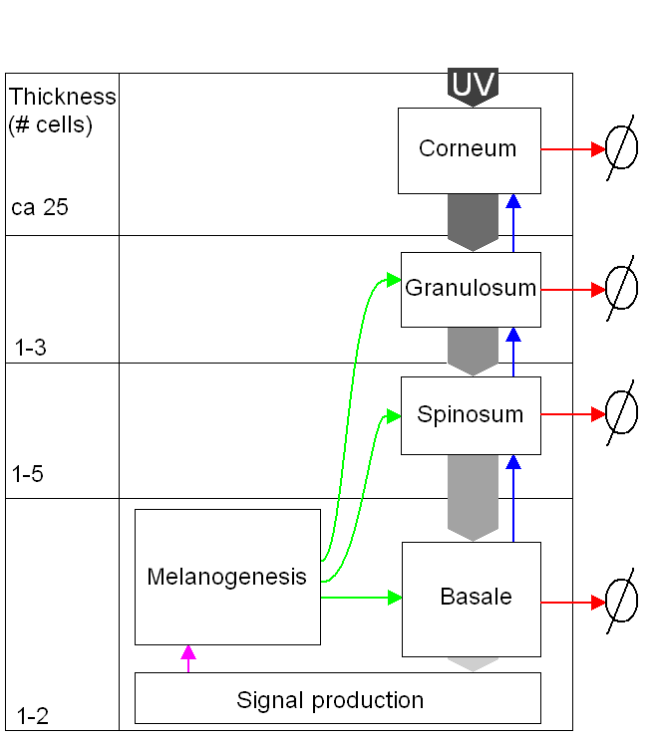
**Melanin production and distribution as response to UV radiation**. Outline of the melanin unit. The melanin content of each layer is a function of melanin delivered from the melanocyte (green arrows), melanin degradation (red arrows) and the melanin in the cells moving upwards (blue arrows). The distributed melanin absorbs UV radiation (described by decreasing darkness of the arrows with increasing depth). Increase of the UV radiation reaching the basal layer triggers signal substance production. In turn, the signal substances stimulate melanogenesis and dendrite growth in the melanocyte (pink arrow).

Here we provide a simple first-generation mathematical model describing the dynamics of melanin content in epidermal layers when the skin is exposed to UV radiation. The main rationale for this effort is to provide a theoretical foundation of appropriate resolution to guide the establishment of a theoretical-experimental research programme aimed at resolving key issues concerning the regulatory anatomy of the tanning response. A conceptual model outlining the major premises underlying the mathematical model is given in Figure 1, and in the remaining part of this section we provide a biological backdrop and the basic premises underlying our current mathematical conceptualization of the tanning phenomenon.

### The tanning response

The tanning response is the additional production and distribution of melanin, exceeding the constitutive level, following UV stimulation. The UV signal is transduced from the primary recipient to the melanocyte, where the photoprotective pigment melanin is produced and distributed. In addition to the optical shielding effects, melanin and its precursors and intermediates act as free-radical scavengers as well as signalling molecules [8-10]. The tanning response thus encompasses UV sensing, signal transduction, melanogenesis, melanosome mobilization and transfer to keratinocytes as well as the further distribution through the epidermis via keratinocyte migration.

### Photobiology of the UV radiation

UV radiation is electromagnetic radiation with wavelengths just below visual light (100–400 nm). The biologically most relevant wavelength segments are UVA (320–400 nm) and UVB (290–320 nm). UVB represents the most bio-reactive part of the spectrum both as inducer of erythema and tanning. Our current conception is that UV radiation causes basal cell skin cancers, such as basal cell carcinoma and malignant melanoma, through its mutagenic effect on basal layer cells. Melanin has a remarkable capacity to absorb UV radiation and to reflect it at the shortest wavelengths (<300 nm) [11].

### Signal transduction

UV radiation is the major inducer of the tanning response. Even though the identities of the primary UV-responsive agents are unknown, both keratinocytes and melanocytes produce various substances that enhance melanogenesis upon UV stimulation [6,12,13]. These comprise the elements controlling the activity of the hypothalamus-pituitary-adrenal axis which are expressed in the skin, including corticotropin releasing hormone (CRH), urocortin, and POMC, with its products ACTH, β- and γ-LPH, CLIP, α- and β-MSH, and β-endorphin [14-16]. The skin is therefore conceived to possess a complete stress handling system of which the tanning response is an important part [17]. The hormone αMSH and its receptor MC1R are important mediators of UV induced tanning [6,18-20]. It has recently been proposed that the increased POMC transcription in keratinocytes following UV exposure is p53 dependent [21]. Even though the POMC derivatives do not seem to be crucial in constitutive melanin production in mouse [22,23], genetic variants of their receptor, MC1R, are deemed to be the main determinant of constitutive melanin levels, and also of the tanning ability in humans [18,24,25]. This enigmatic situation substantiates the challenges involved in understanding the intercellular signalling network of the skin.

### Melanogenesis

Melanogenesis, which occurs within discrete cytoplasmic organelles of the melanocyte called melanosomes, is a process where the amino acid tyrosine is converted into melanin pigment. Tyrosinase is regarded as the rate-limiting enzyme in this process. The transcriptional regulation and the posttranslational activation of tyrosinase are the key regulation points for the melanogenesis. Microphthalmia-associated transcription factor (MITF) is a central protein in the transcriptional regulation of tyrosinase and thereby melanogenesis, as demonstrated by its role in Waardenburg syndrome type 2 and Tietz syndrome [26]. The architecture of the MITF promoter region suggests that several transcription factors (like SOX10, PAX3, LEF1/TCF, CREB and ONECUT2) are involved in melanogenesis and the tanning response [3,5,27]. LEF1/TCF and CREB are responsible for the immensity of MITF promoter responsiveness to UV radiation via MC1R/cAMP/PKA and the WNT/β-Catenin pathways [3,5,28-30]. MITF is therefore proposed to act as a self-regulating switchboard for diverse pathways originating in the cell membrane or the intracellular environment and regulating the activity of the melanogenic apparatus [5]. Other transcription factors can also activate melanin production, like the ubiquitous basic helix-loop-helix-leucine zipper transcription factor USF1, which is reported to be essential for the tanning response, and indeed MITF and USF1 share binding site specificity [31].

Both p38, ERK1/2 and other MAP kinases are involved in MITF signalling and regulation [3,5,32]. The MAP kinase pathway is in turn activated by several ligands such as KITLG, FGF2 and EGF. The MAPK pathway may also activate CREB via RSK1, illustrating the complexity of the structure in these networks. The key second messenger cAMP appears to be another point of cross talk between the αMSH-MC1R pathway and the MAP kinase pathway [33]. See [3-5] for comprehensive reviews of the molecular biology of melanogenesis.

### The melanin is delivered to nearby keratinocytes through dendrites

An additional effect of UV irradiation and the subsequent release of CRH, the POMC derivatives and even endothelins and nitric oxide is stimulation of melanocyte dendrite growth and melanosome delivery to keratinocytes [5,20,33-36]. Each melanocyte attached to the epidermal basement membrane exports mature melanosomes to nearby keratinocytes through its dendrites. The uptake of melanosomes by the keratinocytes is an active process involving regulatory processes in the dendrites as well as in the keratinocytes [8,16,37-39].

### Further distribution through keratinocyte movement

95% of the cells in the epidermis are keratinocytes and a fraction of the keratinocytes in the basal layer is "stem" keratinocytes which produce new keratinocytes continuously through cell division. From being attached to the epidermal basement membrane initially, these "non-stem" keratinocytes move progressively toward the skin surface. In a cross section of the epidermis the keratinocytes form four distinguishable layers named stratum basale, stratum spinosum, stratum granulosum and stratum corneum [40]. The thicknesses of the viable parts of the epidermis (basale, spinosum, granulosum) and of stratum corneum vary between individuals and are correlated with a number of factors like age, body site, gender, UV-exposure, smoking habit, and physical tire [41].

### Mathematical modelling of pigment distribution and production

The model aims to translate the available empirical knowledge into a dynamic model of pigment production and distribution in order to establish a theoretical foundation enabling a deeper and more quantitative understanding of the highly interesting and important tanning phenomenon. The model is described in detail in the Methods section, but here we state its most important premises. The model is designed to describe the dynamics associated with what is called a melanin unit, which consists of one melanocyte and the keratinocytes with which it maintains functional contact. In one melanin unit there are around 36 keratinocytes distributed between the three viable layers [40].

The model describes the melanin content *M*_*j *_in layer *j*, *j *∈ {c, g, s, b} (layers are referred to by their initial letter), the signal substance concentration *s *and the dendrite length relative to the length from mid-melanocyte to stratum corneum, *x*. In the constitutive condition, i.e. in the absence of UV radiation, the melanin produced in the melanocyte is assumed delivered only to the basal layer. The melanin is then distributed outwards by the continuous movement of keratinocytes towards the skin surface. The constitutive level of melanin production and delivery is in equilibrium with the melanin degradation and the loss of melanin through keratinocytes shed from the skin surface. Exposure to UV radiation triggers signal substance production, which, in turn, leads to enhanced melanogenic activity. Due to the resolution level of our model, the complex signal processing is condensed into one signal concentration value *s*, which alters the ratio of the number of ligand bound receptors to the total number of receptors on the melanocyte membrane. While this is a severe simplification of a complex phenomenon, we are still able to capture the systemic behaviour on the current level of resolution. Dendrite growth and melanin production are both assumed to depend on this ratio. Consistent with how biological compounds are normally degraded and in accordance with common modelling practice, degradation of melanin and signal substance are assumed to be linear.

The dendritic growth following UV exposure may show a quite complex geometry, and in the model this growth process has been very much simplified. To derive the functions which describe how melanin is distributed into the epidermal layers, we assume a uniform growth process and that all keratinocytes that can be reached from one melanocyte with a dendrite of a given length will receive the same amount of melanin.

### The available data

Even though a considerable amount of empirical data has been used as a basis for model construction, the amount of available relevant validation data is very modest. To the best of our knowledge, there is currently only one experimental study reported where human skin exposed to UV-exposure is subsequently biopsied to analyse the melanin content in the different epidermal layers [42,43]. In this work, Tadokoro *et al*. exposed nine individuals to a single 1 minimal erythema dose (MED) of UVA/UVB radiation. Biopsies were taken before and seven days after the exposure. Using the Fontana-Masson method, they established the melanin content in the basal, spinosum and granulosum layers of epidermis on these two time points. These data points are reported as single measurements, i.e. no standard error or standard deviation are given. The amount of melanin is given in a not scaled unit and with no measurement of volume. The thicknesses of the different epidermal layers are varying a great deal and it is therefore difficult to establish good concentration measures from these data.

## Results

### Reproduction of empirical data

**Figure 2:**
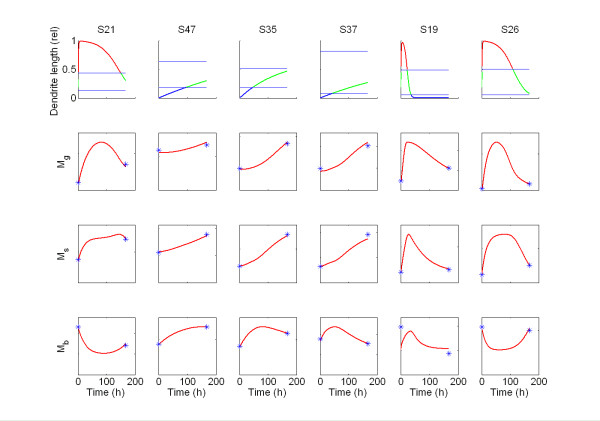
**Temporal evolution of dendrite length and melanin levels in epidermal layers after a pulse of UV radiation**. Each column corresponds to individuals whose melanin data can be described by our model. Top panels: Length of the dendrites relative to total layer thickness. The horizontal lines define the boundaries between the layers. The colour of the curve indicates which layer the tip of the dendrite extends to (blue; basal, green; spinosum, red; granulosum). Lower panels: Temporal evolution of the melanin level in each layer, blue stars indicate observed data. Both in the experiment and the model the UV-pulse was given immediately after the first measurement. In plots displaying melanin levels, scaling is omitted to highlight the fitting of the model to data.

The model was parameterized to fit available data on the distribution of melanin in the different epidermal layers and how different skin types respond to UV radiation. Consistent with Tadokoro *et al*.'s [42] experiments, the model was exposed to a UV pulse. The eight free parameters of the model were optimized to fit nine different data sets representing nine different individuals (S5, S30, S21, S27, S47, S35, S37, S19, S26, using the denotation of Tadekoro *et al.*). For six of the individuals (S21, S47, S35, S37, S19, S26), the model successfully describes the observed melanin distributions and can be calibrated to mimic individual differences (Table 1 and Figure 2, lower panels). The model is not able to describe data corresponding to the three remaining individuals (S5, S30 and S27, see Discussion). Results for these individuals are therefore not shown in the table or the figure.

**Table 1:** Goodness of fit

layer	day	S21	S47	S35	S37	S19	S26
granulosum	0	0.001	0.000	0.001	0.003	0.231	0.006
spinosum	0	0.005	0.000	0.000	0.001	0.007	0.004
basal	0	0.000	0.004	0.003	0.018	0.000	0.002
granulosum	7	0.002	0.000	0.000	0.000	0.048	0.002
spinosum	7	0.000	0.001	0.001	0.008	0.003	0.001
basal	7	0.007	0.003	0.001	0.006	0.000	0.001

sum		0.016	0.008	0.006	0.037	0.290	0.015

The goodness-of-fit ((*M*_data _- *M*_model_)/*M*_data_)^2^, where *M*_data _and *M*_model _are the measured data and model prediction, respectively, for each individual whose data can be successfully described by the model in the three epidermal layers at times *t *= 0 d and *t *= 7 d.

### Dendricity

After the UV pulse, the dendrites first grow and then retract (Figure 2, top panels). The dendrite extension into the layers of epidermis differs substantially between individuals both in length and duration. Individuals S47, S35 and S37 are described by the model with intermediate dendrite lengths lasting longer than the one week simulation time, while S21, S19 and S26 exhibit longer dendrites over a shorter period of time.

### Identifiability

The primary object of the model fitting process is to demonstrate that the model is capable of describing the melanin dynamics in the individuals that constitute our dataset. However, in this context it is important to consider to which degree the actual parameters can be uniquely determined. Such determination may be prevented by the structure of the model equations as such (lack of *structural identifiability*) or, more generally, because the model structure and/or the scarcity or poor quality of the data make it impossible to obtain reliable parameter estimates (lack of *practical identifiability*) [44]. We examined the practical identifiability of the model by assessing parameter determinability in the six individuals which the model could explain. Using the criterion that a parameter is determinable if the absolute value of its cross-correlation with all other parameters is below 0.95, none of the individuals showed determinability in all parameters and no parameter was determinable in all individuals (Table 2). In fact, in individual S26, not a single parameter was deemed to be determinable (see Methods for further details and for information on parameter confidence intervals).

**Table 2:** Determinable parameters

	*f*_min_	γ_M_	ω_b_	ω_g_	*f*_ind_	γ_s_	*a*	*A*
S21			**X**		**X**	**X**	**X**	**X**
S47						**X**		
S35		**X**						**X**
S37							**X**	
S19	**X**	**X**	**X**	**X**	**X**	**X**		
S26								

The checked parameters are determinable for the given individuals using the requirement for determinability given in inequality (22).

### Sensitivity analysis

**Figure 3:**
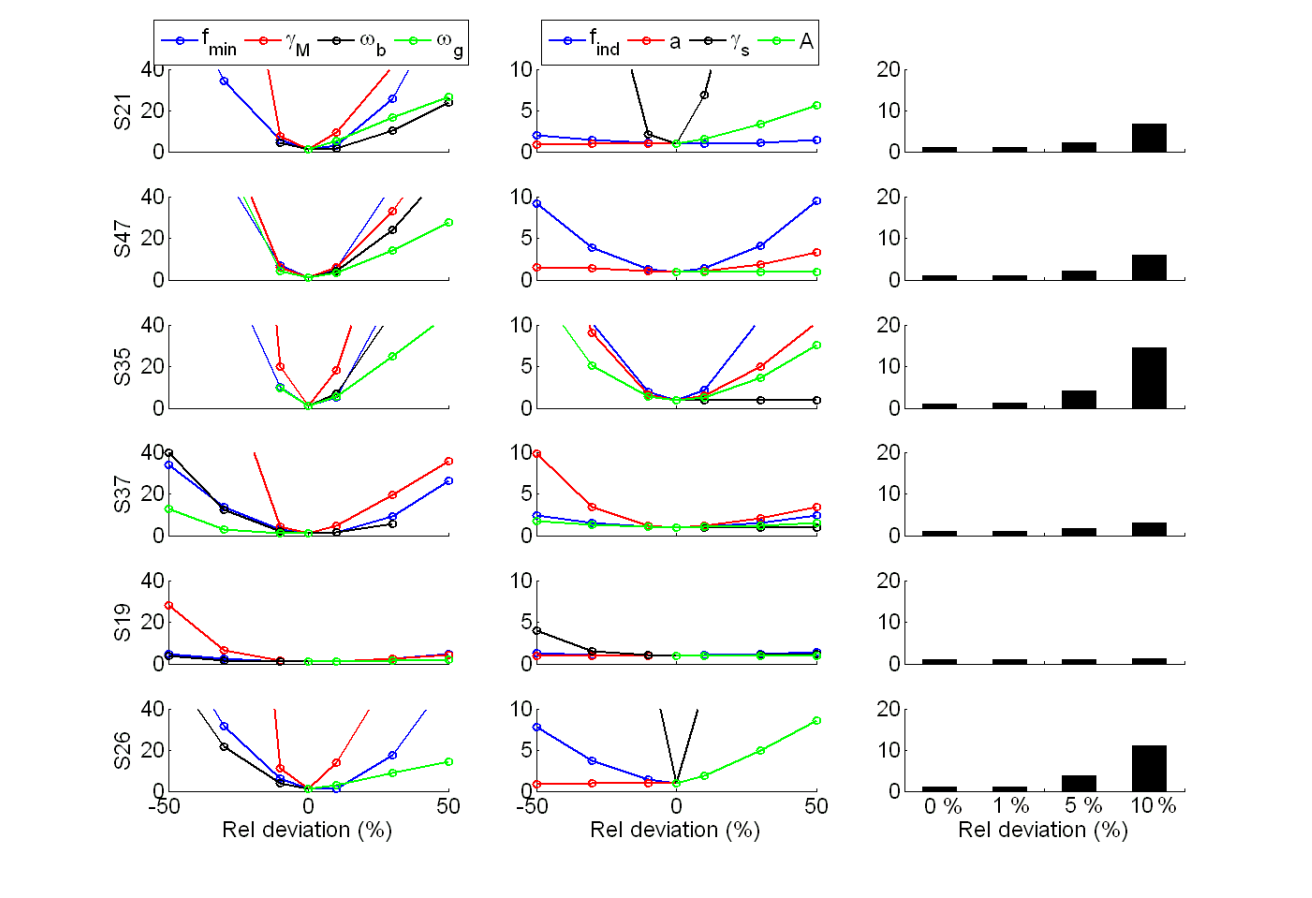
**Sensitivity analysis**. Sensitivity (goodness-of-fit with one perturbed parameter relative to the goodness-of-fit at the optimized parameter set) to variation in the value of one parameter at a time in the neighbourhood of the optimized parameter set, sorted in one high sensitivity group (*f*_min_, γ_s_, ω_s_, ω_b_, left column) and one low sensitivity group (*f*_ind_, *a*, γ_s_, *A*, middle column). The right column shows the mean value of relative change in goodness of fit when all parameter values are subjected to simultaneous random variation 100 times within ranges of 1%, 5% and 10% of the original value.

A sensitivity analysis of the model was performed in order to identify the robustness to variation in each parameter. The sensitivity was assayed by perturbing one parameter value at a time and then computing the goodness of fit for the resulting parameter set relative to the goodness-of-fit of the optimized parameter set as a function of degree of perturbation (Figure 3, left and middle columns). Generally, we observe that the model fit is substantially more sensitive to variation in the parameters *f*_min_, γ_*s*_, ω_*g*_, ω_*b *_than in the parameters *f*_ind_, α, γ_*s*_, *A *(compare the left and middle columns of Figure 3, where the effect of perturbations in these two subsets are displayed, respectively). In addition, for each of the six individual cases we tested to what degree a modest simultaneous random perturbation of all parameters worsened the fit to the empirical data (Figure 3, right column). This exercise reveals that in some individuals (S21, S47, S35 and S26) the fit is substantially worsened with a relatively slight perturbation of the parameter values. These are the same individuals that have a very sharply defined optimum (see corresponding left and middle columns). In comparison, for individuals that have a widely defined optimum (e.g. S37 and S19), the goodness-of-fit is almost not affected by the small random perturbation (right column).

## Discussion

Due to the paucity of relevant experimental data the model is intentionally very simple. Despite this it is capable of making quite strong predictions concerning the relationship between observed temporal development of melanin production and distribution following UV-exposure and the thicknesses of the epidermal layers as well as the degree of dendricity.

The resolution level of the model is a trade-off between the benefits and disadvantages of simple models compared to complex models containing a large amount of molecular detail. In this work we do not explicitly model different signal substances, different receptors (and their genetic variants or their refractory periods), different second messengers and all the actors in the different pathways leading to melanogenesis and dendrification as well as the biochemical regulatory effects of melanin. This does not mean that the model is in conflict with the available information concerning these processes. Given more spatiotemporal experimental data on the tanning response as suggested by the current work, we think the stage will be set for the making of more high-resolution models.

In the six individuals which the model successfully describes, the model fails to achieve parameter determinability due to the wide range of parameter values that are able to provide a fit to data that is almost as good as the best (illustrated in Figure 3). To overcome the poor parameter determinability more data points are needed, given that the model is structurally identifiable. Ideally, future experiments should be designed through collaboration between theoreticians and experimentalists, e.g. by following the rules of identifiability analysis [44,45] to ensure that modellers have maximal use of the data by optimizing the reliability of parameter estimates.

Tadokoro *et al*. [42] provide measurements of three additional individuals for which the model is not capable of achieving consistency with the empirical data. The reason for this is that the melanin levels in the granulosum or spinosum layers in these three individuals are lower 7 days after than before UV-exposure, and the model does not include mechanisms that can produce such behaviour. A possible explanation can be that the UV-exposure causes alteration of the thicknesses of the epidermal layers. A thinner spinosum or granulosum 7 days after UV radiation can, even with a higher relative melanin density, result in a decrease in measured melanin levels with the methods applied by Tadokoro *et al*. [42]. It should be stressed that such a mechanism can easily be implemented so that the model can account for all 9 individuals, but no insight is gained by this exercise before more empirical data become available. In fact, the above discrepancy between model results and empirical data clearly documents the need for including specific measurements of the thickness of epidermal layers in experimental set ups like that of Tadokoro *et al*. [42].

The model does not take into account the possibility that DNA damage due to UV-exposure causes the production of signals stimulating melanogenesis long after the cessation of UV-exposure (reviewed in [46]). Neither does it take into account that the thickness of stratum corneum may change with UV-exposure [47] and that this may influence the UV absorbance [11]. Since, so far, the data on how DNA damage might enhance and prolong signal substance production is so scarce, we find it premature to include this effect in our model. Moreover, the time window of UV radiation in the experiments used to validate our model is probably not long enough for the thickening of stratum corneum to take place and give an effect. Thus, we do not have data to assess whether or not the UV-induced thickening of stratum corneum plays an important role in the tanning process. This does not mean that the above phenomena are not interesting, but that, in the lack of quantitative data, not much would be gained by including them in the model.

## Conclusion

We have translated the available knowledge of the constitutive melanin production and distribution as well as the tanning process in human skin into a differential equations model. The model provides a first generation theoretical framework for a quantitative understanding of the factors underlying observed phenotypic variation in skin colour and tanning ability. In six out of nine individuals for which empirical data exist, the model describes the tanning dynamics and identifies the thickness of the epidermal layers and the degree of dendrification as potentially important sources of variation. The model fails to describe the tanning dynamics in the last three individuals, but by doing so it identifies which data are needed for making an empirically validated improvement of the model to also handle these cases.

## Methods

The aim of the model is to describe the temporal distribution of melanin in the epidermal layers following an UV pulse. Whenever b, s, g or c is used as subscripts below, they refer to the epidermal layers stratum basale, stratum spinosum, stratum granulosum, and stratum corneum, respectively. See Table 3 for an overview of parameters and their dimensions.

**Table 3:** Parameters of the model with approximate magnitudes, references, descriptions and units

Parameter	Appr. value	Reference	Description	Unit
γ_s_	10^-1^	Optimized from data	Signal substance degradation rate	1/h
*f*_min_	10^3^	Optimized from data	Constitutive melanin production	tau/h
*f*_ind_	10^3^	Optimized from data	Tanning ability	tau/h
*A*	10^0^	Optimized from data	Dendrite growth rate	cd/h
γ_M_	10^-2^	Optimized from data	Melanin degradation rate	1/h
*w*	10^-1^	Derived from [40]	Cell movement rate	cell/h
*T*_c_	25	Derived from [40]	Thickness, corneum	cd
*T*_g_	2	Derived from [40]	Thickness, granulosum	cd
*T*_s_	4	Derived from [40]	Thickness, spinosum	cd
*T*_b_	1	Derived from [40]	Thickness, basale	cd
*area*	5	Derived from [40,42,48]	Area of melanin unit	cd*cd

Unit of volume is number of cells and unit of length is cell diameter (cd). Melanin amount is measured in the arbitrary unit of [42] (tau). Estimated values are related to each other and the rest of the model. Their absolute values are not regarded as predictions.

### UV intensity and signal substance dynamics

The spatial intensity of the UV pulse is assumed to decrease exponentially with depth and absorbance down through the epidermis. As increased melanin content enhances UV absorption, the net signal substance production rate relative to baseline conditions as a function of UV intensity *I *and melanin content *M* may be expressed as:

(1)

We assume that there is a maximal production rate *a*_1 _and that the actual production is a sigmoidal function of *I *with threshold parameter Θ (i.e. the production rate is half of its maximum when *I *equals Θ). In the exponential term, *M*_0 _is the constitutive melanin level and *M *is the current total melanin level in all layers; . In the simulations, since the melanin amount in the epidermis does not change significantly during the first hour, and the model is exposed to UV radiation only during this period, the exponential term can be regarded as a constant. Furthermore, the UV intensity is assumed constant during the one hour of exposure and therefore the fraction term can be regarded as a constant as well. In the simulations the production rate can thus be regarded to be constant: .

The concentration of free signal substance is determined by the balance between its production and degradation, described by

(2)

where we assume linear degradation with relative rate γ_*s *_and that the pulse of signal substance production (with magnitude ) endures for only one hour, i.e. the ODE to be solved is

(3)

The analytical solution of equation (3) is

(4)

Let *R *stand for the fractional occupancy of melanocyte receptors binding signal substance. Then the rate of change of *R *is given by *k*_+_*s*(1-*R*) - *k*_*R*, where *k*_+_*s *and *k*_ are coupling and decoupling relative rates, respectively. We assume that an equilibrium between binding and release of signal molecules from the melanocyte surface is set up so fast relative to the time scale of the model that the rate of change can be set equal to zero, giving *R *in terms of signal substance concentration, i.e.

(5)

where *K *= *k*_/*k*_+_. Thus,

(6)

where .

### Melanin production

Melanocytes have a constitutive melanin production as well as the ability to increase melanin production as a response to receptor mediated signals. The melanin synthesis rate can thus be expressed as

(7)

In the absence of UV radiation the net signal substance level (not including base line levels) and hence the net fractional occupancy, *R*, is zero, such that *f*_min _expresses the constitutive melanin production.

### Dynamics of dendrite length

We assume that all the melanin is delivered to nearby keratinocytes. The melanin delivered to one particular layer is described as the production *f*(*R*) times a function *d*_*j *_for each layer *j*, where the *d*_*j*_'s are functions of the dendrite length relative to the maximum dendrite length, here denoted *x*. The dendrites grow when the melanocyte is stimulated by signal substances [5,33]. We therefore assume that *x *is governed by

(8)

where the parameter *a *is the time constant of dendrite growth and retraction. Assuming that the dendrite is of zero extension initially, equation (8) can be analytically solved to give

(9)

### Distribution of melanin as function of dendrite length

**Figure 4:**
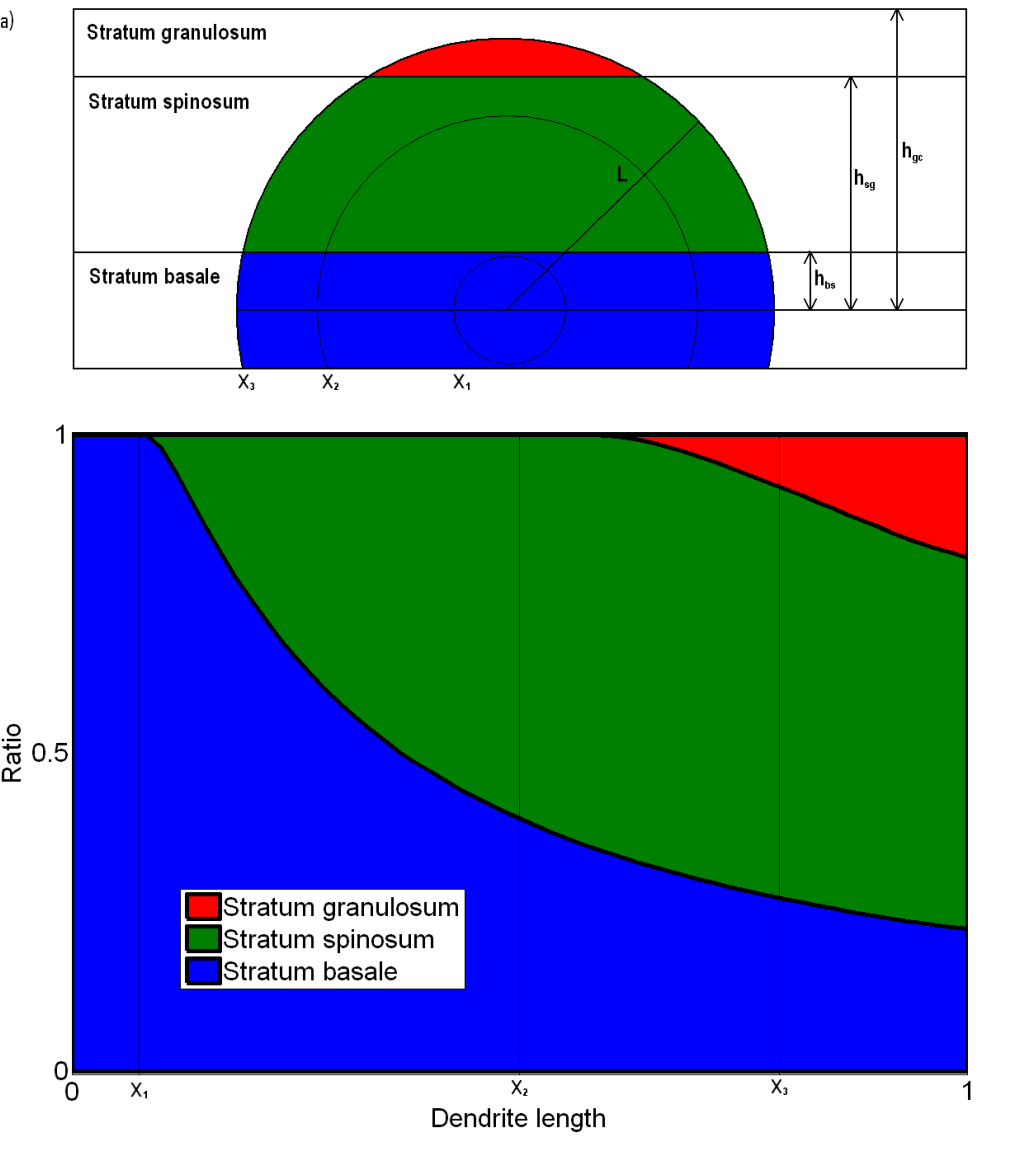
**Melanin distribution ratios between the different layers as a function of the dendrite length**. As the length of the dendrite, *x*, grows, the ratio of reached volume in each layer changes (a). In b) these ratios are plotted versus the dendrite length. With short dendrites (*x*_1_) all melanin is delivered to the basal layer. As the dendrite is growing, more is delivered to stratum spinosum (*x*_2_), and when they are long enough they also distribute melanin to the stratum granulosum (*x*_3_).

Assume a sphere with the melanocyte in the middle and radius equal to the dendrite length *x *(Figure 4a). The proportion of this sphere that resides in each layer of the epidermis is equated with the proportion of melanin delivered to this layer. As the radius of the sphere grows, the proportion of the volume residing in each layer changes. We use these proportions as guidelines for distributing the melanin produced. The proportion functions below (10) derived from the formula for volume of a sphere and the thickness of the different layers are plotted in Figure 4b. We define all dendrite tips to be in the basal layer provided that the relative dendrite length *x *is below *h*_*bs*_, where *h*_bs _= 1/2*T*_b_/(1/2*T*_b _+ *T*_s _+ *T*_g_) (*T*_*j *_denotes thickness of layer *j*) is the distance from the centre of the melanocyte to the border between stratum basale and stratum spinosum relative to the maximal dendrite length which equals 1/2*T*_b _+ *T*_s _+ *T*_g_. The dendrite tips extend to the spinosum layer when *h*_bs _≤ *x *≤ *h*_sg _and to the granulosum layer when *x *> *h*_sg_, where *h*_sg _= *h*_bs _+ *T*_s_/(1/2*T*_b _+ *T*_s _+ *T*_g_) is the distance from the centre of the melanocyte to the border between stratum spinosum and stratum granulosum relative to 1/2*T*_b _+ *T*_s _+ *T*_g_. We then obtain the functions *d*_*j*_, *j *= b, s, g, describing the melanin delivery distributions,

(10)

It should be noted that *d*_g_(*x*) + *d*_s_(*x*) + *d*_b_(*x*) = 1 holds for all *x*.

### Melanin dynamics within keratinocytes

The data obtained by Tadokoro *et al*. [42] are given in an absolute but not scaled unit of the amount of melanin in each layer. Using the same unit, we model the melanin content in each layer by the following set of differential equations, where *M*_*j *_denotes melanin content in layer *j*,

(11)

where the terms *d*_*j*_(*x*)*f*(*R*), *j *= b, s, g, represent melanin delivery rates from melanocytic dendrites into keratinocytes of layer *j *and ω_*j *_= *w*/*V*_*j*_, *j *= b, s, g, c (*w *and *V*_*j *_are described below). We assume that melanin has a relative degradation rate γ_M _(with unit *h*^-1^) and that *w *(unit: cell *h*^-1^) describes the speed by which keratinocytes move towards the surface. The scope of this model is the melanin unit (defined in Background and not to be confused with a unit of measurement) which we assume to have the same area throughout the epidermis (described by the parameter *area*). The volume of each layer is then *V*_*j *_= *T*_*j *_× *area*.

With the above definition of ω_*j*_, *j *= b, s, g, the quantities *h*_bs _and *h*_sg _can be defined in terms of the model parameters as follows;

(12)

The sum of the inverses of the ω_*j*_'s, *j *= b, s, g, equals (*T*_b_/2 + *T*_s _+ *T*_g_) × *area*/*w*, which is approximately 400 (Table 3). Thus, if ω_b _and ω_g _are given, ω_s _follows by a simple calculation.

### Estimates of parameter ranges

The parameters of the model and their values are presented in Table 3. In the following we describe the derivations of parameter value ranges.

### Volume of the layers, *V*

The volume of each layer is given as the number of cells in one melanin unit. In the simulations we have divided the volume into thickness *T*_*j*_, *j *= c, g, s, b and *area*. In this model the melanin unit has a fixed area throughout the epidermis, hence only the thickness varies between the layers. The layer thicknesses, presented in Table 3, are obtained from [40]. The melanocyte density, and thus the area of one melanin unit, does not vary between different skin colours but it does vary with body location [40]. Melanocyte density is measured in different ways, but from [40,42,48] we have derived an average of the area of the melanin unit on the actual body site (lower back) to be 4–6 cells.

### Cell movement, *w*

The parameter *w *(with unit cell per hr) describes the movement of keratinocytes upwards from layer to layer. The epidermal turnover time *t*_turnover_, the time from a cell is born in the basal layer until it is shred off the corneum layer, is 52 – 75 days in normal skin [40]. The parameter *w *can then be estimated from the relation

(13)

where *T *is the total thickness of all four layers.

### Parameter estimation using a maximum-likelihood approach

The model parameters were fitted to data provided by [42] which describe the melanin amount in the three lowest layers of epidermis before and 7 days after UV irradiation in nine individuals. We denote the 9 individual data sets according to the codes used by Tadokoro *et al*. [42,43].

Analytical solution formulas for the variables in equation (11) are obtained by solving the equation for *M*_b _given *x*(*t*) and *R*(*t*), then solving the equation for *M*_s _given *M*_b_(t) and, finally, using *M*_s_(*t*) to solve the equation for *M*_g_(*t*). We thus obtain the solution formulas

(14)

We note that for *M*_b _there is an accumulation of melanin due to delivery (*d*_b_) whereas for *M*_s _and *M*_g _in addition to the delivery term there is a term due to the outwardly directed melanin transport.

By setting the derivatives equal to zero we find expressions for the steady state values of the variables of the model. Under baseline conditions the melanin production is at its minimum and the dendricity is zero. Hence the melanin contents are simply determined by setting the derivatives in equation (11) equal to zero and setting *d*_*g*_(*x*) = 0, *d*_*s*_(*x*) = 0, *d*_*b*_(*x*) = 1, and *f*(*R*) = *f*(0) = *f*_min_. Then

(15)

In the same manner as above *M*_c _can be found, but we omit the calculation of the corresponding solution formula as this variable is not needed in the parameter estimation.

In order to obtain robust parameter estimates, we used a maximum likelihood approach where one seeks to maximize the so-called likelihood function (the probability density of a model for the occurrence of the measurements given a set of parameters). Assuming the probability of the measurements to be uncorrelated normal distributions, the log-likelihood function is

(16)

where *N *is the number of measurements  with variance  and *M*_*k*_(**p**) is the model prediction given the vector of parameters **p**. If we assume the measurement noise to be Gaussian with variance that is proportional to the magnitude of the measurement with proportionality *f *(which is reasonable since Tadekoro *et al. *count pixels in a picture), minimizing the log-likelihood function is equivalent to least squares minimization of the quantity

(17)

with respect to the parameters **p**. Note that *f *is unknown since we do not have information on the statistics of the pixel counting process. Thus, in terms of our model and data we should minimize

(18)

where  and , *j *= b, s, g, are the melanin data in the basal, spinosum and granulosum layers at 0 and 7 days, respectively,  and  are the model predictions at 0 and 7 days, given in equations (15) and (14) respectively, as function of the parameter vector **p **which is defined as

(19)

With the exception of *f*_min_, we enforce constraints on all the parameters;

(20)

The constraints on ω_g _and ω_b _are due to empirical data (calculated from Table 3).

The remaining constraints were chosen to ensure that the model predicts an effect of the UV pulse. For example, without the constraint γ_s _> 0 the minimization routine frequently suggested that γ_s _is zero in the proposed minimum. In consequence, the fractional occupancy of melanocyte receptor is constant, meaning that melanin synthesis beyond the baseline level is not induced.

In the minimization algorithm the function *x*(*t*) and the functions *M*_*j*_(*t*), *j *= b, s, g, evaluated in *t *= 7 × 24 h, are computed using the trapezoid method with Δ*t *= 1 for numerical computation of the integrals in equations (15) and (14), respectively. The constrained minimization routine fmincon (Mathworks Inc.) using an active-set algorithm is applied to determine the parameter values that give the best fit of the model to empirical data.

### Practical identifiability

With the present model and data we have more unknowns than observations, i.e. a negative number of degrees of freedom. This makes it impossible to perform a rigorous statistical treatment of the model goodness-of-fit and of the parameter significance, which would otherwise be a natural part of practical identifiability analysis. We therefore focus our analysis on parameter determinability.

The simplest way of assessing parameter determinability is by computing the cross-correlation between the different parameters [44]. The cross-correlation between parameters *i *and *j *is

(21)

where *V*_*ij *_are the entries of the estimated parameter covariance matrix  (defined below). A large correlation between two parameter estimates (close to -1 or 1) indicates that the two parameters are weakly determinable because of their large influence on each other. We will require that

(22)

for parameter *i *to be considered determinable.

Taking the second partial derivatives of the log-likelihood function *L *with respect to the parameter vector **p **shows that the information matrix is closely related to the Hessian of *F*

(23)

From this an estimate of the parameter error covariance matrix can be obtained;

(24)

For each individual which the model successfully describes, the Hessian of *F *evaluated in the minimum is computed, giving the estimated parameter covariance matrix from which the parameter cross-correlations are obtained using equation (21) (given in additional file 1: Doc1). In individual S26 all parameters show correlations at 1 or -1 (due to the information matrix being very close to singular) indicating that none of the parameters are determinable. In the remaining individuals, some, but not all, parameters are determinable (Table 2 and additional file 1: Doc1).

Using the Cramer-Rao bound for multivariate data for the *i*'th parameter (as before, IM denotes the information matrix and (IM^-1^)_*ii *_denotes the *i*'th diagonal element of its inverse),

(25)

we can use the diagonal elements of the parameter covariance matrix to produce the confidence interval for parameter *i*;

(26)

The quantity  is proportional to the unknown factor *f*. In order for the estimated confidence intervals not to include zero, *f *must be sufficiently small. In Additional file 1: Doc1 are listed all estimated parameter confidence intervals expressed in terms of *f *for all individuals that the model is able to describe and the corresponding inequality that *f *must satisfy in order that the confidence interval does not include zero. We observe that in four of the individuals (S47, S35, S37 and S26) *f *must be unrealistically small (of order 10^-4 ^or less) and in two individuals (S19 and S21) the constraints are much less strict (of order 10^-2^) for the confidence intervals to not include zero. Interestingly, individuals S19 and S21 exhibit the most consistent confidence intervals as well as the largest number of determinable parameters (Table 2).

## Authors' contributions

SWO and EH conceived the study. JT carried out the model development, simulations and manuscript drafting. LØ participated in the model development and in the manuscript drafting. EH contributed in the parameterization of the model, provided biological understanding of the problem, and participated in the manuscript drafting. SWO participated in the study design and coordination and helped to draft the manuscript. All authors read and approved the final manuscript.

## Supplementary Material

Additional file 1 **Estimated parameter covariance matrices and confidence intervals**. Matlab output of estimated parameter cross-correlation matrices (Vhat) and parameter confidence intervals (CI) expressed by the ratio of measurement noise to measurement magnitude for the individuals which the model explains. Click here for file
