# Supplementary material for: The mathematics of tanning
Source: BMC Syst Biol. 2009 Jun 9;3:60. doi: 10.1186/1752-0509-3-60 (PMC2714304; doi:10.1186/1752-0509-3-60)
Supplement: Additional file 1 — Estimated parameter covariance matrices and confidence intervals. Matlab output of estimated parameter cross-correlation matrices (Vhat) and parameter confidence intervals (CI) expressed by the ratio of measurement noise to measurement magnitude for the individuals which the model explains. [file 1752-0509-3-60-S1.pdf]

****

'S21'

Vhat =

1.0000	0.9793	0.8734	-0.9308	0.3354	-0.4920	0.1353	0.0665
0.9793	1.0000	0.9352	-0.9737	0.4740	-0.5055	0.1921	0.0389
0.8734	0.9352	1.0000	-0.9491	0.6474	-0.3779	0.3511	-0.0410
-0.9308	-0.9737	-0.9491	1.0000	-0.5191	0.5638	-0.1372	-0.1083
0.3354	0.4740	0.6474	-0.5191	1.0000	0.0207	0.3750	-0.0335
-0.4920	-0.5055	-0.3779	0.5638	0.0207	1.0000	0.2707	-0.1279
0.1353	0.1921	0.3511	-0.1372	0.3750	0.2707	1.0000	-0.8880
0.0665	0.0389	-0.0410	-0.1083	-0.0335	-0.1279	-0.8880	1.0000

CI fmin   : (440.6253 - 1.96f x 1629.8302, 440.6253 + 1.96f x 1629.8302), f < 0.13793
CI gamma_m: (0.026635 - 1.96f x 0.099952, 0.026635 + 1.96f x 0.099952), f < 0.13596
CI omega_b: (0.011089 - 1.96f x 0.042965, 0.011089 + 1.96f x 0.042965), f < 0.13168
CI omega_g: (0.005 - 1.96f x 0.015333, 0.005 + 1.96f x 0.015333), f < 0.16638
CI find   : (112.1677 - 1.96f x 1165.8997, 112.1677 + 1.96f x 1165.8997), f < 0.049085
CI gamma_s: (1 - 1.96f x 21.261, 1 + 1.96f x 21.261), f < 0.023997
CI a   : (0.032638 - 1.96f x 0.1304, 0.032638 + 1.96f x 0.1304), f < 0.1277
CI A   : (0.01 - 1.96f x 0.16174, 0.01 + 1.96f x 0.16174), f < 0.031545
****

'S47'

Vhat =

1.0000	0.9999	-0.9924	-0.9999	-1.0000	0.8240	0.9991	-0.9998
0.9999	1.0000	-0.9939	-1.0000	-0.9999	0.8301	0.9994	-1.0000
-0.9924	-0.9939	1.0000	0.9936	0.9925	-0.8799	-0.9947	0.9943
-0.9999	-1.0000	0.9936	1.0000	0.9999	-0.8286	-0.9993	1.0000
-1.0000	-0.9999	0.9925	0.9999	1.0000	-0.8244	-0.9991	0.9998
0.8240	0.8301	-0.8799	-0.8286	-0.8244	1.0000	0.8352	-0.8316
0.9991	0.9994	-0.9947	-0.9993	-0.9991	0.8352	1.0000	-0.9995
-0.9998	-1.0000	0.9943	1.0000	0.9998	-0.8316	-0.9995	1.0000

CI fmin   : (230.6257 - 1.96f x 42958.2979, 230.6257 + 1.96f x 42958.2979), f < 0.0027391
CI gamma_m: (0.0053836 - 1.96f x 3.7178, 0.0053836 + 1.96f x 3.7178), f < 0.0007388
CI omega_b: (0.008 - 1.96f x 0.20923, 0.008 + 1.96f x 0.20923), f < 0.019508
CI omega_g: (0.008125 - 1.96f x 2.6331, 0.008125 + 1.96f x 2.6331), f < 0.0015744
CI find   : (160.9399 - 1.96f x 11176.1459, 160.9399 + 1.96f x 11176.1459), f < 0.0073471
CI gamma_s: (0.0021651 - 1.96f x 0.042478, 0.0021651 + 1.96f x 0.042478), f < 0.026005
CI a   : (1e-05 - 1.96f x 7.1228, 1e-05 + 1.96f x 7.1228), f < 7.163e-07
CI A   : (0.01 - 1.96f x 785.8867, 0.01 + 1.96f x 785.8867), f < 6.4921e-06

****

'S35'

Vhat =

1.0000	-0.1895	0.1111	-0.1526	-0.9839	0.1338	0.1225	-0.1856
-0.1895	1.0000	-0.5749	0.7709	0.1931	-0.7113	-0.6515	0.9210
0.1111	-0.5749	1.0000	-0.9566	-0.1132	0.8259	0.9236	-0.6617
-0.1526	0.7709	-0.9566	1.0000	0.1555	-0.8792	-0.9304	0.8311
-0.9839	0.1931	-0.1132	0.1555	1.0000	-0.1363	-0.1248	0.1891
0.1338	-0.7113	0.8259	-0.8792	-0.1363	1.0000	0.9762	-0.7711
0.1225	-0.6515	0.9236	-0.9304	-0.1248	0.9762	1.0000	-0.7286
-0.1856	0.9210	-0.6617	0.8311	0.1891	-0.7711	-0.7286	1.0000

CI fmin   : (398.5181 - 1.96f x 0.0069949, 398.5181 + 1.96f x 0.0069949), f < 29067.8582
CI gamma_m: (0.016618 - 1.96f x 0.00068091, 0.016618 + 1.96f x 0.00068091), f < 12.4517
CI omega_b: (0.008 - 1.96f x 0.079282, 0.008 + 1.96f x 0.079282), f < 0.051482
CI omega_g: (0.0061795 - 1.96f x 0.050206, 0.0061795 + 1.96f x 0.050206), f < 0.062798
CI find   : (500.6826 - 1.96f x 0.00060906, 500.6826 + 1.96f x 0.00060906), f < 419420.1874
CI gamma_s: (0.006877 - 1.96f x 0.42317, 0.006877 + 1.96f x 0.42317), f < 0.0082914
CI a   : (1e-05 - 1.96f x 1.6462, 1e-05 + 1.96f x 1.6462), f < 3.0994e-06
CI A   : (0.46077 - 1.96f x 0.077996, 0.46077 + 1.96f x 0.077996), f < 3.0141
****

'S37'

Vhat =

1.0000	0.9965	0.9779	-0.9855	0.9994	-0.9533	0.2468	-0.9084
0.9965	1.0000	0.9885	-0.9920	0.9972	-0.9694	0.2370	-0.9260
0.9779	0.9885	1.0000	-0.9808	0.9808	-0.9853	0.2423	-0.9407
-0.9855	-0.9920	-0.9808	1.0000	-0.9856	0.9618	-0.2431	0.9119
0.9994	0.9972	0.9808	-0.9856	1.0000	-0.9609	0.2576	-0.9204
-0.9533	-0.9694	-0.9853	0.9618	-0.9609	1.0000	-0.2734	0.9787
0.2468	0.2370	0.2423	-0.2431	0.2576	-0.2734	1.0000	-0.3788
-0.9084	-0.9260	-0.9407	0.9119	-0.9204	0.9787	-0.3788	1.0000

CI fmin   : (1030.7349 - 1.96f x 8669.4124, 1030.7349 + 1.96f x 8669.4124), f < 0.06066
CI gamma_m: (0.040746 - 1.96f x 0.34499, 0.040746 + 1.96f x 0.34499), f < 0.06026
CI omega_b: (0.017066 - 1.96f x 0.13736, 0.017066 + 1.96f x 0.13736), f < 0.063392
CI omega_g: (0.01458 - 1.96f x 0.22515, 0.01458 + 1.96f x 0.22515), f < 0.033039
CI find   : (508.4534 - 1.96f x 92.7114, 508.4534 + 1.96f x 92.7114), f < 2.7981
CI gamma_s: (0.0027048 - 1.96f x 0.059996, 0.0027048 + 1.96f x 0.059996), f < 0.023002
CI a   : (1e-05 - 1.96f x 0.039544, 1e-05 + 1.96f x 0.039544), f < 0.00012902
CI A   : (0.35096 - 1.96f x 11.7628, 0.35096 + 1.96f x 11.7628), f < 0.015223

```
****************************************************************

    'S19'

Vhat =

    1.0000   -0.3840   -0.7822   -0.7791    0.9072    0.0854   -0.1929    0.3914
   -0.3840    1.0000    0.4407    0.3790   -0.4338   -0.0561    0.1594   -0.2538
   -0.7822    0.4407    1.0000    0.7717   -0.8887   -0.1165    0.5172   -0.6426
   -0.7791    0.3790    0.7717    1.0000   -0.8546   -0.0764    0.3374   -0.5145
    0.9072   -0.4338   -0.8887   -0.8546    1.0000    0.1052   -0.3426    0.5121
    0.0854   -0.0561   -0.1165   -0.0764    0.1052    1.0000    0.0217   -0.0349
   -0.1929    0.1594    0.5172    0.3374   -0.3426    0.0217    1.0000   -0.9527
    0.3914   -0.2538   -0.6426   -0.5145    0.5121   -0.0349   -0.9527    1.0000

CI fmin   : (38.6486 - 1.96f x 52.7454, 38.6486 + 1.96f x 52.7454), f < 0.37385
CI gamma_m: (0.012368 - 1.96f x 0.00097425, 0.012368 + 1.96f x 0.00097425), f < 6.4768
CI omega_b: (0.024 - 1.96f x 0.052375, 0.024 + 1.96f x 0.052375), f < 0.23379
CI omega_g: (0.0051511 - 1.96f x 0.0061525, 0.0051511 + 1.96f x 0.0061525), f < 0.42716
CI find   : (500.5009 - 1.96f x 7.7344, 500.5009 + 1.96f x 7.7344), f < 33.0156
CI gamma_s: (1 - 1.96f x 4.0073, 1 + 1.96f x 4.0073), f < 0.12732
CI a      : (0.20852 - 1.96f x 1.3014, 0.20852 + 1.96f x 1.3014), f < 0.081752
CI A      : (0.01 - 1.96f x 0.30529, 0.01 + 1.96f x 0.30529), f < 0.016712
****************************************************************

    'S26'

Vhat =

    1.0000   -1.0000   -1.0000    1.0000   -1.0000   -1.0000    1.0000   -1.0000
   -1.0000    1.0000    1.0000   -1.0000    1.0000    1.0000   -1.0000    1.0000
   -1.0000    1.0000    1.0000   -1.0000    1.0000    1.0000   -1.0000    1.0000
    1.0000   -1.0000   -1.0000    1.0000   -1.0000   -1.0000    1.0000   -1.0000
   -1.0000    1.0000    1.0000   -1.0000    1.0000    1.0000   -1.0000    1.0000
   -1.0000    1.0000    1.0000   -1.0000    1.0000    1.0000   -1.0000    1.0000
    1.0000   -1.0000   -1.0000    1.0000   -1.0000   -1.0000    1.0000   -1.0000
   -1.0000    1.0000    1.0000   -1.0000    1.0000    1.0000   -1.0000    1.0000

CI fmin   : (403.3484 - 1.96f x 20525.1741, 403.3484 + 1.96f x 20525.1741), f < 0.010026
CI gamma_m: (0.051181 - 1.96f x 688.3771, 0.051181 + 1.96f x 688.3771), f < 3.7934e-05
CI omega_b: (0.023642 - 1.96f x 655.5134, 0.023642 + 1.96f x 655.5134), f < 1.8402e-05
CI omega_g: (0.0053521 - 1.96f x 173.4406, 0.0053521 + 1.96f x 173.4406), f < 1.5744e-05
CI find   : (920.6241 - 1.96f x 175155080.8603, 920.6241 + 1.96f x 175155080.8603), f < 2.6817e-06
CI gamma_s: (1 - 1.96f x 442251.5678, 1 + 1.96f x 442251.5678), f < 1.1537e-06
CI a      : (0.042322 - 1.96f x 8692.7423, 0.042322 + 1.96f x 8692.7423), f < 2.484e-06
CI A      : (0.01 - 1.96f x 13980.1216, 0.01 + 1.96f x 13980.1216), f < 3.6495e-07
```
